# A retrospective study on antibacterial treatments for koalas infected with *Chlamydia pecorum*

**DOI:** 10.1038/s41598-023-39832-w

**Published:** 2023-08-04

**Authors:** Chien-Jung Chen, Andrea Casteriano, Alexandra Clare Green, Merran Govendir

**Affiliations:** Sydney School of Veterinary Science, The University of Sydney, Sydney, NSW Australia

**Keywords:** Ecological epidemiology, Infectious-disease epidemiology

## Abstract

Chlamydiosis remains the leading infectious disease and is one of the key factors responsible for the dramatic reduction of koala populations in South-East Queensland (SEQ) and New South Wales (NSW) regions of Australia. Possible infection outcomes include blindness, infertility, painful cystitis, and death if left untreated. Studies have reported the treatment efficacy of chloramphenicol and doxycycline, which are the two most commonly administered treatments in diseased koalas, in clinical settings. However, none have directly compared the treatment efficacy of these antibacterials on koala survival. A retrospective study was essential to identify any relationships between the demographical information, and the animals’ responses to the current treatment regimens. Associations were explored between six explanatory (sex; maturity; location; clinical signs, treatment; treatment duration) and two outcome variables (survival; post-treatment PCR). Results showed that female koalas had a statistical trend of lower odds of surviving when compared to males (OR = 0.36, *p* = 0.05). Koalas treated with chloramphenicol for ≥ 28 days had greater odds of surviving than when treated for < 28 days (OR = 8.8, *p* = 0.02), and those koalas administered doxycycline had greater odds of testing PCR negative when compared to chloramphenicol treatments (OR = 5.45, *p* = 0.008). There was no difference between the antibacterial treatments (chloramphenicol, doxycycline, and mixed/other) and the survival of koalas. Female koalas had greater odds of exhibiting UGT signs only (OR = 4.86, *p* < 0.001), and also greater odds of having both ocular and UGT clinical signs (OR = 5.29, *p* < 0.001) when compared to males. Of the koalas, 28.5% initially had no clinical signs but were PCR positive for *C. pecorum.* This study enables further understanding of the complex nature between chlamydial infection and response to antibacterial treatment.

## Introduction

Chlamydiosis is the leading infectious disease affecting the majority of wild koala populations in South East Queensland (SEQ) and New South Wales (NSW) in Australia^1^. *Chlamydia pecorum*, the bacterial pathogen primarily responsible for chlamydiosis in koalas^2^, infects the conjunctiva resulting in conjunctivitis, and/or infects the urogenital and reproductive tracts (UGT)^3,4^. Blindness, infertility, severe, painful cystitis, and death are just some of the possible infection outcomes if left untreated^5,6^.

Enrofloxacin^7^ and chloramphenicol^8,9^ have been the two major systemically administered antibacterials to treat chlamydiosis in the koala over the last two decades, but since the withdrawal of the injectable chloramphenicol formulations from the Australian veterinary market in 2013 and 2014^10^, other antibacterial treatments such as weekly doxycycline injections have been introduced^11^. While studies demonstrated that enrofloxacin alone was incapable of reaching therapeutic targets at a safe dosage, via oral or subcutaneous routes^7,12,13^, both chloramphenicol and doxycycline treatments have resulted in improvement in clinical signs ^11^.

Longitudinal studies of the efficacy of treatments of infected koalas have been reported from localised geographical regions, however, the study period of the most recent were only up to 2017^14,15^ and 2018^16^, and none have directly compared the treatment efficacy of chloramphenicol and doxycycline on koala survival. The Koala Health Hub (KHH) is an initiative of The University of Sydney and provides a service to wildlife veterinarians Australia-wide, to diagnose *C. pecorum* infections in koalas by polymerase chain reaction (PCR) from swabs of both eyes and the UGT. Information about the patient submitted to the KHH with the swabs frequently contains demographic information, therapeutic treatments course/s administered by the wildlife hospitals, and the outcome of many infected koalas (survived/died). The quantitative PCR results for *C. pecorum* were obtained by the KHH from the swabs collected. Therefore, the aim of this study was to conduct a retrospective study on the KHH records from 2017 – 2020 to identify any relationships between the infected koalas and various geographical locations, ages, sex, and the animals’ responses to the current treatments, to enable further understanding of the complex nature between chlamydial infection and response to antibacterial treatment.

## Methods

### Animals and study sites

Information accompanying swabs from koalas that had clinical signs of chlamydiosis, or were suspected to have chlamydiosis, were submitted to the KHH from some wildlife hospitals and zoos in Queensland (QLD), NSW, South Australia (SA), and Western Australia (WA). The data in this study were retrieved from the 2017 – 2020 KHH database. For each koala, swabs were usually collected from the conjunctiva (ocular) site of both eyes, and/or the UGT at the cloaca or penile urethra when the presence of chlamydial infection was suspected. Post-treatment swabs, for PCR analysis, were also frequently collected two weeks after the treatment course to detect the presence of chlamydial pathogens. PCR, targeting *C. pecorum,* was performed by following the procedures and conditions previously described^17^.

### Data collection

Data were collected on the koalas’ demographical information, including: animal identification (ID), sex (male or female), maturity (juvenile [< 2 years of age], young adult [2–4 years of age], middle-aged [5–7 years of age], or senior [≥ 8 years of age]) determined by the severity of the wear on the premolar and molar teeth^18^, location (NSW, QLD, SA or WA), clinical signs (no clinical signs, ocular signs only, UGT signs only, or both ocular and UGT signs), and the outcome (released, permanent captive, or euthanised).

The antibacterial treatments used (chloramphenicol, doxycycline, or mixed/other) and the number of days for the initial treatment (and which also applies to subsequent treatments if applicable) were also recorded and are summarised in Table 1. Attempts to follow up any incomplete data for each koala were made by contacting the treating hospital directly three months apart, on two occasions.

**Table 1:** Summary of the initial treatment regimens administered to koalas with chlamydial infections.

Antibacterial	Concentration (mg/mL)	Dosage (mg/kg)	Frequency of administration	Route
Chloramphenicol (manufacturer Ceva)^1^	150	60	Once daily	Subcutaneous injection
Doxycycline (manufacturer Vetafarm)^2^	50	5	Every seven days	Subcutaneous injection
Mixed/other^3^	Variable	Variable	Variable	Variable

^1^ = Ceva, Glenorie, NSW: 14–45 days, ready to use. ^2^ = Vetafarm, Wagga Wagga, NSW: 4–42 days, diluted at 50:50 in saline. ^3^ = 3–42 days, may include systemic enrofloxacin, polymyxin B sulphate, carprofen injection and/or various topical ointments such as tetracycline eye ointment applied to the conjunctiva and cornea.

Koalas with information on the treatment regimen (i.e., antibacterials and dosage, and days in treatment) were included for the treatment analyses and the demographic description.

### Data management and cleaning

The dataset was saved in Microsoft Excel® in a comma-separated values (.csv) format and imported into R studio version 1.4.1717, an integrated development environment in R^19^ for data cleaning and organisation. The variables that were re-categorised included: the outcome variable from ‘released’, and ‘permanent captive’ to ‘survived’, and euthanised’ to ‘died’; the location variable where ‘SA’ and ‘WA’ was combined to ‘other’; the treatment duration variable which was re-categorised to ‘ < 28 days’ and ‘ ≥ 28 days’; and the ‘PCR eyes’ and ‘PCR UGT’ variables merged into ‘PCR post’ (yes/no). The post-treatment PCR results of ocular and UGT swabs were merged into positive or negative, irrespective of the site of infection. The treatment duration categories of 28 days were determined due to the recommended treatment regimen^20,21^. This data cleaning process was achieved using the tidyverse^22^ and janitor packages^23^. All subsequent data manipulations and analyses were performed in jamovi^24^.

Two outcome variables, namely the outcome (1 = survived/0 = died) and the post-treatment PCR results (1 = positive/0 = negative) at either the ocular or UGT sites, were explored against six explanatory variables. Of which, three were binary categorical variables including sex (male/female), maturity (juvenile/adult [≥ 2 years of age]), and treatment duration (< 28 days/ ≥ 28 days); and three were multi-categorical variables comprising clinical signs (no clinical signs; ocular signs only; UGT signs only; both ocular and UGT signs), location (NSW/QLD/other), and treatment (chloramphenicol; doxycycline; mixed/other).

### Descriptive analyses

For individual categorical variables, counts and percentages were examined to explore distribution across the categories.

### Inferential analyses

Univariable binary logistic regression models were formulated to examine the individual associations between the six categorical explanatory variables and two outcome variables. Further, for the analyses on the initial treatment course, data were stratified according to the treatment (chloramphenicol; doxycycline; mixed/other). For the 2 × 2 associations between treatment duration and the outcome (survived/died) for each treatment, Fisher’s exact tests were conducted due to low counts in some categories. When there were observations of zero for a given category, a constant of one was added to each of the categories to maintain its initial proportions to obtain the odds ratios (OR) and p-values. However, raw counts for each category are reported in the results. For all statistical analyses, the level of significance was set at *p* < 0.05,with values between *p* < 0.1 to 0.05 considered a statistical trend.

## Results

### Descriptive analyses

#### Koala demographics

A total of 177 koalas were observed in this study with the descriptive results presented in Table 2. There was a relatively even spread of 51.4% males and 48.6% females, and of the koalas with age recorded, 10.1% were juveniles, and 89.1% were adults. Most koalas were from NSW wildlife hospitals (68.9%), followed by QLD (16.4%), and other states (14.7%). A total of 123 koalas displayed clinical signs associated with chlamydiosis, of which 48% had ocular signs such as conjunctivitis, 35.8% had UGT signs such as cystitis and a urine-stained rump, and 16.3% had signs from both sites. A further 28.5% of the koalas had no clinical signs but tested positive for *C. pecorum* (65.3% survived, 4.1% died, and for 30.6% the outcome was unspecified).

**Table 2:** Counts and percentages of sex, maturity, location, clinical signs and treatments of infected koalas between 2017 and 2020.

Variable	Category	Count	Percentage
Sex	Male	90	51.4
	Female	85	48.6
	Total	175	100
Maturity	Juvenile (< 2 yrs)	17	10.1
	Adult (≥ 2yrs)	151	89.9
	Total	168	100
Location	NSW	122	68.9
	QLD	29	16.4
	Other	26	14.7
	Total	177	100
Clinical signs	No clinical signs	49	28.5
	Ocular signs only	59	34.3
	UGT signs only	44	25.6
	Ocular and UGT signs	20	11.6
	Total	172	100
Initial treatment	Chloramphenicol	78	50.3
	Doxycycline	66	42.6
	Mixed/other	11	7.1
	Total	155	100

Not all variables have total counts of 177 due to unknowns in some categories.

#### Initial antibacterial treatment course administered

Excluding the koalas without treatments recorded (12.4%), approximately half of the koalas were initially treated with daily chloramphenicol subcutaneous injections (50.3%), and the remaining treated with either weekly subcutaneous doxycycline injections (42.6%) or mixed/other treatments (7.1%) (Table 2).

The once-daily chloramphenicol injections (60 mg/kg) were the most frequently administered treatment (47.1% of all treatments) with 11.7% of chloramphenicol treatments administered < 28 days, 68.8% of chloramphenicol treatments administered for ≥ 28 days, and for 19.5% the treatment duration was unknown. Weekly subcutaneous doxycycline injections (5 mg/kg) (46.3% of all treatments) were administered with 39.4% of doxycycline treatments for < 28 days, 47.0% of doxycycline treatments for ≥ 28 days, and 13.6% with an unknown treatment duration. The remaining koalas (6.6% of all treatments) were administered drug combinations or other antibacterial treatments (i.e., systemic enrofloxacin, polymyxin B sulphate, carprofen injection and/or various topical ointments such as tetracycline eye ointment applied to the cornea/conjunctiva) as the initial treatment.

#### Outcome of the koalas

A total of 19 koalas (10.7%) died, irrespective of the number of times treated. After the initial treatment, 16 of these 19 koalas were euthanised including four administered chloramphenicol for < 28 days, three administered with chloramphenicol ≥ 28 days, two with doxycycline for < 28 days, four treated with doxycycline for ≥ 28 days, and three with mixed/other treatments (Table s S1–S4).

A further total of 19 koalas (10.7%) had more than one course of treatment, of these, four koalas had a total of three or more treatments (Table 3). Of these 19, three were euthanised after the second treatment, including two which received chloramphenicol for 28 days, followed by 21 days of chloramphenicol for one, and the other administered with mixed/other for seven days. The third euthanised koala was treated with doxycycline for 21 days, then again for 28 days. The survival percentage of koalas with a single treatment was 60.8% (regardless of the treatment regimen), as opposed to 63.2% for the re-treated koalas.

**Table 3:** Treatment summary of re-treated koalas and their survival outcome.

No	Sex	Age	Maturity	Clinical sign	Location	Initial treatment	Initial treatment duration (days)	Second treatment	Second treatment duration (days)	Third treatment	Third treatment duration (days)	Outcome
1	F	1	Juvenile	Ocular only	NSW	Chloramphenicol	≥ 28	Chloramphenicol	< 28			Died
2	M	6	Adult	Both ocular and UGT	QLD	Chloramphenicol	≥ 28	Chloramphenicol	< 28			
3	M	4	Adult	UGT only	NSW	Chloramphenicol		Chloramphenicol				
4	F	9	Adult	UGT only	NSW	Chloramphenicol	≥ 28	T.N.R				Died
5	M	5	Adult	No clinical sign	NSW	Chloramphenicol	≥ 28	T.N.R				Survived
6	M	4	Adult	UGT only	NSW	Chloramphenicol	≥ 28	Chloramphenicol	< 28			Survived
7	F	5	Adult	UGT only	NSW	Doxycycline	< 28	Doxycycline	≥ 28			Died
8	F	11	Adult	UGT only	QLD	Chloramphenicol	≥ 28	Chloramphenicol	≥ 28			Survived
9	F	11	Adult	UGT only	QLD	Doxycycline	≥ 28	Chloramphenicol	≥ 28			Survived
10	M	4	Adult	Both ocular and UGT	NSW	Chloramphenicol	≥ 28	Mixed/other	< 28			Died
11	M	1	Juvenile	Ocular only	NSW	Doxycycline	< 28	Doxycycline	< 28			Survived
12	F	15	Adult	No clinical sign	NSW	Doxycycline	< 28	Doxycycline	< 28			Survived
13	F	1	Juvenile	Ocular only	QLD	Chloramphenicol	≥ 28	Chloramphenicol	< 28			Survived
14	F	5	Adult	Both ocular and UGT	NSW	Chloramphenicol	≥ 28	Doxycycline	< 28	Doxycycline	≥ 28	Survived
15*	M	2	Adult	Ocular only	NSW	Chloramphenicol	< 28	Chloramphenicol		Chloramphenicol		
16	M	2	Adult	UGT only	NSW	Mixed/other	< 28	Chloramphenicol	< 28			Survived
17	F	1	Juvenile	UGT only	NSW	Mixed/other	< 28	Chloramphenicol	< 28			Survived
18	M	5	Adult	Both ocular and UGT	NSW	Mixed/other	< 28	Chloramphenicol	< 28	Mixed/other	< 28	Survived
19*	F			Both ocular and UGT	NSW	Chloramphenicol	≥ 28	Mixed/other	< 28	Mixed/other	< 28	Survived

* = koala received more than three treatments. T.N.R = treatment not recorded.

The remaining koalas (n = 158) were either released (n = 102), remained captive for further monitoring (n = 6), or their fate was unspecified (n = 50).

The data summary table for each year cohort of infected koalas is provided in supplementary Tables S1–S4, as well as the raw data of each koala categorised by year in Tables S5–S8.

### Inferential statistics

#### Association between the demographic variables and survival of koalas

The univariable association between the sex, maturity, location, clinical signs, and initial treatment of the animals to their survival is provided in Table 4. There was a statistical trend between sex of koalas and the outcome (survived/died), with female koalas having lower odds of surviving in comparison to males (OR = 0.36, *p* = 0.05). No association was found between the other variables and survival of the koalas.

**Table 4:** Univariable results showing the factors affecting the survival of koalas after the initial treatment administration using binomial logistic regression.

Variable	Category	Survived (%)	Died (%)	Odds ratio (95% CI)	*p* value
Sex	Male ^1^	60 (47.6)	6 (4.8)	1.00	**0.05**
	Female	47 (37.3)	13 (10.3)	0.36 (0.13, 1.02)	
	Total	107 (84.9)	19 (15.1)		
Maturity	Juvenile ^1^	14 (11.2)	2 (1.6)	1.00	0.75
	Adult	92 (73.6)	17 (13.6)	0.77 (0.16, 3.71)	
	Total	106 (84.8)	19 (15.2)		
Location	NSW ^1^	75 (59.1)	16 (12.6)	1.00	0.36
	QLD	25 (19.7)	2 (1.6)	2.67 (0.57, 12.42)	
	Other	8 (6.3)	1 (0.8)	1.71 (0.20, 14.62)	
	Total	108 (85.0)	19 (15.0)		
Clinical signs	No clinical signs ^1^	32 (25.6)	2 (1.6)	1.00	0.23
	Ocular clinical signs only	38 (30.4)	7 (5.6)	0.34 (0.07, 1.75)	
	UGT clinical signs only	26 (20.8)	7 (5.6)	0.23 (0.04, 1.21)	
	Ocular and UGT signs	10 (8.0)	3 (2.4)	0.21 (0.03, 1.43)	
	Total	106 (84.8)	19 (15.2)		
Initial treatment	Chloramphenicol ^1^	51 (40.8)	9 (7.2)	1.00	0.34
	Doxycycline	49 (39.2)	7 (5.6)	1.24 (0.43, 3.58)	
	Mixed/other	6 (4.8)	3 (2.4)	0.35 (0.07, 1.67)	
	Total	106 (84.8)	19 (15.2)		

CI = confidence interval. ^1^ = reference category.

Statistical trend value is in [bold].

#### Association between the initial treatment courses and survival of koalas

Of the koalas that were administered chloramphenicol as the initial treatment, there was a significant difference in survival for the various treatment durations. Koalas treated for ≥ 28 days had higher odds of surviving than when treated for < 28 days (OR = 8.8, *p* = 0.02) (Table 5). However, there was no difference between the treatments (chloramphenicol versus doxycycline) and the survival of the koalas. Further, no association between the duration of treatment and the survival of koalas for doxycycline and mixed/other treatments were identified.

**Table 5:** Univariable association between the duration of the initial treatment course and survival of the koalas for chloramphenicol, doxycycline, and mixed/other treatments using Fisher’s exact test.

Treatment	Duration (days)	Median duration (range)	Survived (%)	Died (%)	Odds ratio (95% CI)	*p* value
Chloramphenicol	< 28 ^1^	14 (14–25)	4 (7.0)	4 (7.0)	1.00	**0.02**
	≥ 28	28 (28–45)	44 (77.2)	5 (8.8)	8.8 (1.67, 46.57)	
	Total		48 (84.2)	9 (15.8)		
Doxycycline	< 28 ^1^	21 (4–21)	23 (41.1)	3 (5.4)	1.00	1.00
	≥ 28	28 (28–42)	26 (46.4)	4 (7.1)	0.85 (0.17, 4.19)	
	Total		49 (87.5)	7 (12.5)		
Mixed/other	< 28 ^1^	14 (3–24)	3 (37.5)	0 (0)	1.00	0.30
	≥ 28	34 (30–42)	2 (25.0)	3 (37.5)	0.19 (0.01, 2.66)	
	Total		5 (62.5)	3 (37.5)		

CI = confidence interval. Koalas with unknown antibacterials, treatment duration, and/or outcomes were excluded from this table. ^1^ = reference category.

Significant value is in [bold].

#### Association between the demographic variables and the post-treatment PCR after the initial treatment course

There was an association between the treatment type and the post-treatment PCR results, irrespective of the site of infection (Table 6). The koalas administered doxycycline treatments had greater odds of testing PCR negative at any site post-treatment in comparison to koalas injected with chloramphenicol (OR = 5.45, *p* = 0.008). There was no significant difference between sex, maturity, location, or clinical signs of koalas with post-treatment PCR results.

**Table 6:** Univariable results showing the factors affecting post-treatment PCR after the initial treatment course using binomial logistic regression.

Variable	Category	Post-treatment PCR		Odds ratio (95% CI)	*p* value
		Positive (%)	Negative (%)		
Sex	Male ^1^	10 (9.9)	41 (40.6)	1.0	0.84
	Female	9 (8.9)	41 (40.6)	1.11 (0.41, 3.02)	
	Total	19 (18.8)	82 (81.2)		
Maturity	Juvenile^1^	3 (3.0)	9 (8.9)	1.0	0.57
	Adult	16 (15.8)	73 (72.3)	1.52 (0.37, 6.26)	
	Total	19 (18.8)	82 (81.2)		
Location	NSW^1^	15 (14.6)	51 (49.5)	1.0	0.22
	QLD	4 (3.9)	16 (15.5)	1.18 (0.34, 4.06)	
	Other	1 (1.0)	16 (15.5)	4.71 (0.58, 38.46)	
	Total	20 (19.4)	83 (80.6)		
Clinical signs	No clinical signs ^1^	2 (1.9)	25 (24.3)	1.0	0.14
	Ocular signs only	7 (6.8)	31 (30.1)	0.35 (0.07, 1.86)	
	UGT signs only	7 (6.8)	20 (19.4)	0.23 (0.04, 1.22)	
	Ocular and UGT signs	4 (3.9)	7 (6.8)	0.14 (0.02, 0.93)	
	Total	20 (19.4)	83 (80.6)		
Treatment	Chloramphenicol^1^	13 (14.0)	31 (33.3)	1.0	**0.008**
	Doxycycline	3 (3.2)	39 (41.9)	5.45 (1.43, 20.84)	
	Mixed/other	3 (3.2)	4 (4.3)	0.56 (0.11, 2.86)	
	Total	19 (20.4)	74 (79.6)		

^1^ = reference category; CI = confidence interval.

Significant value is in [bold].

#### Association between maturity and clinical signs against male and female koalas

Univariable associations were observed between the clinical signs and sex (Table 7). The results suggest that female koalas have greater odds of having UGT signs only (OR = 4.86), and greater odds of having both ocular and UGT clinical signs (OR = 5.29, *p* < 0.001), in comparison to males.

**Table 7:** Univariable results showing the relationship between maturity, clinical signs, and sex using binomial logistic regression.

Variable	Category	Male (%)	Female (%)	Odds ratio (95% CI)	*p* value
Maturity	Senior ^1^	16 (9.6)	15 (9.0)	1.0	0.11
	Middle-aged	34 (20.5)	17 (10.2)	0.53 (0.21, 1.33)	
	Young adult	30 (18.1)	37 (22.3)	1.32 (0.56, 3.09)	
	Juvenile	8 (4.8)	9 (5.4)	1.20 (0.37, 3.92)	
	Total	88 (53.0)	78 (47.0)		
Clinical signs	No clinical signs^1^	34 (20.0)	15 (8.8)	1.0	** < 0.001**
	Ocular signs only	33 (19.4)	24 (14.1)	1.65 (0.74, 3.68)	
	UGT signs only	14 (8.2)	30 (17.6)	4.86 (2.02, 11.69)	
	Both clinical signs	6 (3.5)	14 (8.2)	5.29 (1.70, 16.42)	
	Total	87 (51.2)	83 (48.8)		

Maturity age groups: juvenile (< 2 yrs old), young adult (2–4 yrs old), middle-aged (5–7 yrs old), senior (≥ 8 yrs old). ^1^ = reference category; CI = confidence interval.

Significant value is in [bold].

## Discussion

This is the first study to investigate the demographic information (sex, age, submitting wildlife hospital location) of 177 infected koalas, along with the treatment efficacy and the survival outcome using objective data. There are similar studies which provide some insights into the epidemiology of chlamydial infections in wild koalas^25^, disease progression^14^, and there are also prospective, unblinded treatment trials of antibacterials, such as chloramphenicol injections (60 mg/kg, *s.c.* once daily for 28 days)^11,20^, doxycycline injections (5 mg/kg, *s.c./i.m.* every seven days for 28 days), and enrofloxacin injections (10 mg/kg then 5 mg/kg, *s.c.* once daily for 28 days)^11^. However, investigating the associations between the demographical information and the animals’ responses to various treatments allows a better understanding of the disease interactions in koalas.

The findings demonstrated a statistical trend for female koalas having lower odds of surviving than males. There were similar proportions of male (51.4%) and female (48.6%) koalas in the study, with no association between the maturity and sex. The distribution of males versus females across the age ranges did not significantly differ, which suggest the lower odds of survival were not due to a younger or older female population. Further, the results determined that female koalas had greater odds of having UGT signs only, and greater odds of exhibiting both ocular and UGT clinical signs, compared to males. Therefore, the fact that females have greater odds of contracting UGT infections may account for their possible lower survival rate. On the contrary, other studies have found no association between sex and the survival outcome after treatment^16,20^. This could be due to the differences in the study aims, as Charalambous and Narayan (2020) not only considered koalas with chlamydial infections, but also with poor body condition, trauma, and dehydration^16^. Further observations also align with another study which concluded that koalas with UGT infections were significantly less likely to recover than ocular infections, although infections at both sites encountered the lowest likelihood of recovering^14^. This study also demonstrated that over two-thirds (68.4%) of the re-treated koalas had either UGT only or both ocular and UGT clinical signs. Although some clinical signs are more challenging to treat than others, no correlation was evident between the clinical signs and survival outcome in this study.

The results of this study demonstrate that both ocular and UGT swabs are taken from all koalas when presented to the koala hospital without any obvious clinical signs to screen for sub-clinical chlamydiosis, as 28.5% of the infected koalas (20% males and 8.8% females when excluding the koalas with missing information on sex) had no clinical signs present. Of the koalas with sub-clinical chlamydial infections, 65.3% survived, 4.1% died, and for 30.6% the outcome was unspecified. Possible reasons for this include that koalas were in the early stages of disease development at the time of sampling, or that they had sub-clinical chlamydial infections^26^. Treating koalas with sub-clinical infections can help significantly reduce further transmissions within their populations, as the chlamydial load was reported to be higher in koalas with no clinical signs^25^. This study is not suggesting that these sub-clinical cases should be treated with antibacterials, however, a previous study^14^ observed that most sub-clinical cases (2/3 wild koalas) do eventuate in clinical disease so monitoring these koalas could be advantageous.

Chloramphenicol and doxycycline were the two most widely injected antibacterials during the study period. Assuming that the standard treatment regimen, which incorporates the dosage, dosage interval, and administration route^21^ of chloramphenicol and doxycycline, were consistent as described in Table 1, koalas treated with chloramphenicol for ≥ 28 days (median duration of 28 days) had greater odds of surviving than when koalas were treated with chloramphenicol for < 28 days (median duration of 14 days). This finding is supported by Markey et al., (2007) which administered chloramphenicol for a duration of 45 days at the same dosage in diseased koalas, and successfully eliminated infections from all sites^8^. However, there were no significant differences observed for the duration of doxycycline and the mixed/other treatments.

Irrespective of the site of the post-treatment PCR, an association was identified between chloramphenicol and doxycycline against the post-treatment PCR results. Koalas administered doxycycline had greater odds of testing PCR negative at the end of the treatment course when compared to koalas treated with chloramphenicol. Although there is no association between the treatment type and the survival outcomes of koalas, the findings in this study and Booth and Nyari (2020) suggest that doxycycline may be more effective at reducing the bacterial load than chloramphenicol^11^. Further, there are other possible factors affecting the survival outcome of these animals, such as unknown underlying health conditions, and the side effects of treatments (such as dehydration, weight loss, gut dysbiosis, development of candidiasis, and decrease in body condition)^11^. As a result, an association was evident between the treatment type and post-treatment PCR, but not between the survival outcomes.

Interestingly, 84.2% of the re-treated koalas had either UGT only, or both ocular and UGT clinical signs. However, no differences between the survival rates of treated once only, and re-treated koalas were found. Therefore, further treatments are worthy of consideration if required.

Of the host-associated factors, genetics, and co-infection with immunosuppressive pathogens, such as koala retrovirus (KoRV), are proposed to be heavily associated with chlamydial disease in wild koala populations^27,28^. Altogether, the spread of KoRV may have a significant impact on the prevalence and severity of chlamydiosis in the northern and southern populations whenever co-infections occur. Unfortunately, the koalas were not tested for KoRV in this study. Geographical distribution may also be one of the factors affecting the survival of koalas infected with chlamydiosis. Koalas with closer geographical proximity are expected to share similar gene pools which may vary in disease tolerance and could also influence their responses to various treatments. Anecdotally, observations from wildlife hospitals reported that koalas in different regions have differences in diet and thus, variation in gut microbiota. Variation in chlamydial genotypes may also affect the severity of the clinical signs exhibited. A study by Legione et al., (2016) revealed that genotype B was dominant in Victorian koalas infected with *C. pecorum,* and that this genotype has not been reported in QLD and NSW populations^29^. As Victorian koalas general display milder clinical signs when compared to their QLD and NSW counterparts, the presence of genotype B could indicate that it is less pathogenic^29^. Therefore, location of the koalas was also considered, with 68.9% of the study population from NSW and 16.4% from QLD. However, no association was determined between the location of the koalas, the survival outcome, and post-treatment PCR results. A possible reasoning could be due to the considerable differences between the number of NSW and QLD koalas in this study.

Lack of completeness of the data was a limitation in this study, which reduced the subject number to the current size (n = 177). Due to the low or zero counts in some categories, a constant of one was added to each of the categories to maintain its initial proportions for yielding odds ratios and p-values. To overcome this issue, a simple, unified template could be created to ensure the data of interest are collected, which simplifies record keeping. Another limitation was the differences in animal ID methods. Each hospital has its own animal ID system, so tracking the history of koalas re-admitted to different hospitals was challenging. Setting a universal animal ID system across all the wildlife hospitals can overcome this limitation and improve the accuracy of data for future retrospective studies. Future studies could conduct a longer-term follow up and have data on the severity of the clinical signs and the adverse effects observed for more accurate interpretations of the treatment efficacy. Despite these limitations, the study provides valuable information by successfully highlighting some associations between sex and disease status, and comparative success of the systemic treatments for chlamydiosis. This also provides a foundation for possible future research, such as investigating the long-term post-treatment responses of koalas with varied severity levels of chlamydial infections from detailed clinical records.

This retrospective study on wild koalas treated for chlamydiosis between 2017 and 2020 proposed significant associations, that have not been reported, between initial treatment duration of chloramphenicol and the survival outcome; treatment administered and the post-treatment PCR; and sex and the clinical signs present. Statistical trend between sex and the survival outcome found that female koalas may have lower odds of surviving than males. Further analyses revealed that UGT signs are more common in females than males, however, whether koalas with UGT signs have a lower survival rate remains unknown. As 28.5% of the koalas have sub-clinical chlamydial infections, it is suggested that routine screening should be conducted during health checks, even if no clinical signs are present. In relation to the survival outcome, there is no discernment in the antibacterials administered, however, koalas were more likely to remain PCR positive after the chloramphenicol treatment. Interestingly, animals have a greater chance of survival when treated with chloramphenicol for ≥ 28 days versus for < 28 days. There was no evidence that re-treatments should be discouraged, as this study found no difference in the survival of koalas treated once only versus multiple re-treatments. Based on the findings, developing a standard screening protocol for chlamydial infections in wild koalas can be effective at identifying disease in its early stage, or in koalas without significant clinical signs. This also impresses the need for long-term monitoring of treated koalas to ascertain their survival and reproduction activity after treatment.

### Supplementary Information

Supplementary Information.

## Data Availability

The data presented in this study are available in this article and the Supplementary Information attached.
