# Supplementary material for: A retrospective study on antibacterial treatments for koalas infected with Chlamydia pecorum
Source: Sci Rep. 2023 Aug 4;13:12670. doi: 10.1038/s41598-023-39832-w (PMC10403558; doi:10.1038/s41598-023-39832-w)
Supplement: Supplementary file 1 — Supplementary Information. [file 41598_2023_39832_MOESM1_ESM.docx]

**Supplementary Information**

**Table S1.** Raw data summary of the koalas admitted to wildlife hospitals in 2017.

| **Treated once (n= 35)** | |
| --- | --- |
| Adult (2 yrs+) | 30 |
| Juvenile (<2 yrs old) | 2 |
| Unknown (age) | 3 |
| Sex | M: 14; F: 21 |
| Unknown (sex) | 0 |
| Wild/captive | W: 35; C: 0 |
| Location | NSW: 28; QLD: 6; SA: 1 |
| Clinical signs present | Y: 30 (ocular: 14; UGT: 9; Ocular, UGT: 6; PHX: 1); N: 2; Unknown: 3 |
| Treatment and duration | Chloramphenicol (14d): 1 |
|  | Chloramphenicol (17d): 1 |
|  | Chloramphenicol (28d): 18 |
|  | Chloramphenicol (35d): 1 |
|  | Chloramphenicol (35d), Chlorsig ointment (70d): 1 |
|  | Chloramphenicol (45d): 4 |
|  | Chloramphenicol (unknown): 7 |
|  | Enrofloxacin (14d), chloramphenicol (21d): 1 |
|  | Unknown treatment and duration: 1 |
| PCR results | Chloramphenicol (14d)- negative: 1/1 |
|  | Chloramphenicol (17d)- unknown: 1/1 |
|  | Chloramphenicol (28d)- negative: 17/18; positive: 1/18 |
|  | Chloramphenicol (35d)- suspect: 1/1 |
|  | Chloramphenicol (35d), Chlorsig ointment (70d)- negative: 1/1 |
|  | Chloramphenicol (45d)- negative: 4/4 |
|  | Chloramphenicol (unknown)- negative: 5/7; suspect: 2/7 |
|  | Enrofloxacin (14d), chloramphenicol (21d)- positive: 1/1 |
|  | Unknown treatment and duration- negative: 1/1 |
| Outcomes | Chloramphenicol (14d)- unspecified: 1/1 |
|  | Chloramphenicol (17d)- euthanised: 1/1 |
|  | Chloramphenicol (28d)- released: 17/18; permanent captive: 1/18 |
|  | Chloramphenicol (35d)- unspecified: 1/1 |
|  | Chloramphenicol (35d), Chlorsig ointment (70d)- permanent captive: 1/1 |
|  | Chloramphenicol (45d)- released: 4/4 |
|  | Chloramphenicol (unknown)- released: 1/7; unspecified: 6/7 |
|  | Enrofloxacin (14d), chloramphenicol (21d)- euthanised: 1/1 |
|  | Unknown treatment and duration- permanent captive: 1/1 |
| **Treated twice (n= 6)** | |
| Adult (2 yrs+) | 5 |
| Juvenile (<2 yrs old) | 1 |
| Unknown (age) | 0 |
| Sex | M: 4; F: 2 |
| Unknown (sex) | 0 |
| Wild/captive | W: 6; C: 0 |
| Location | NSW: 5; QLD: 1 |
| Clinical signs present | Y: 5 (ocular: 1; UGT: 3; both: 1); N: 1; Unknown: 0 |
| Treatment and duration | Chloramphenicol (28d), chloramphenicol (7d): 1 |
|  | Chloramphenicol (28d), chloramphenicol (14d): 1 |
|  | Chloramphenicol (28d), chloramphenicol (21d): 1 |
|  | Chloramphenicol (28d), unknown (unknown): 1 |
|  | Chloramphenicol (unknown), chloramphenicol (unknown): 1 |
|  | Chloramphenicol (42d), unknown treatment and duration: 1 |
| PCR results | Chloramphenicol (28d), chloramphenicol (7d)- negative: 1/1 |
|  | Chloramphenicol (28d), chloramphenicol (14d)- negative: 1/1 |
|  | Chloramphenicol (28d), chloramphenicol (21d)- positive: 1/1 |
|  | Chloramphenicol (28d), unknown (unknown)- negative: 1/1 |
|  | Chloramphenicol (unknown), chloramphenicol (unknown)- negative: 1/1 |
|  | Chloramphenicol (42d), unknown treatment and duration- negative: 1/1 |
| Outcomes | Chloramphenicol (28d), chloramphenicol (7d)- released: 1/1 |
|  | Chloramphenicol (28d), chloramphenicol (14d)- unspecified: 1/1 |
|  | Chloramphenicol (28d), chloramphenicol (21d)- euthanised: 1/1 |
|  | Chloramphenicol (28d), unknown (unknown)- euthanised: 1/1 |
|  | Chloramphenicol (unknown), chloramphenicol (unknown)- unspecified: 1/1 |
|  | Chloramphenicol (42d), unknown treatment and duration- released: 1/1 |

**Table S2.** Raw data summary of the koalas admitted to wildlife hospitals in 2018.

| **Treated once (n=40)** | |
| --- | --- |
| Adult (2 yrs+) | 33 |
| Juvenile (<2 yrs old) | 4 |
| Unknown (age) | 3 |
| Sex | M: 24; F: 16 |
| Unknown (sex) | 0 |
| Wild/captive | W: 38; C: 2 |
| Location | NSW: 26; QLD: 6; SA: 7; WA: 1 |
| Clinical signs present | Y: 28 (ocular: 16; UGT: 10; both: 2); N: 12; Unknown: 0 |
| Treatment and duration | Chloramphenicol (14d): 3 |
|  | Chloramphenicol (21d): 1 |
|  | Chloramphenicol (28d): 6 |
|  | Chloramphenicol (unknown): 2 |
|  | Doxycycline (14d): 1 |
|  | Doxycycline (18d): 1 |
|  | Doxycycline (21d): 6 |
|  | Doxycycline (28d): 5 |
|  | Doxycycline (35d): 3 |
|  | Doxycycline (42d): 2 |
|  | Doxycycline (unknown): 2 |
|  | Enrofloxacin (20d), chloramphenicol (22d), chlorsig ointment (17d), terramyacin (32d): 1 |
|  | Enrofloxacin (21d), chloramphenicol (9d), terramyacin (31d): 1 |
|  | Enrofloxacin (21d), chloramphenicol (12d), terramyacin (33d): 1 |
|  | Unknown treatment and duration: 5 |
| PCR results | Chloramphenicol (14d)- negative: 3/3 |
|  | Chloramphenicol (21d)- negative: 1/1 |
|  | Chloramphenicol (28d)- negative: 6/6 |
|  | Chloramphenicol (unknown)- suspect: 1/2; positive: 1/2 |
|  | Doxycycline (14d)- negative: 1/1 |
|  | Doxycycline (18d)- negative: 1/1 |
|  | Doxycycline (21d)- negative: 6/6 |
|  | Doxycycline (28d)- negative: 4/5, positive: 1/5 |
|  | Doxycycline (35d)- negative: 3/3 |
|  | Doxycycline (42d)- positive: 1/2; negative: 1/2 |
|  | Doxycycline (unknown)- negative: 2/2 |
|  | Enrofloxacin (20d), chloramphenicol (22d), chlorsig ointment (17d), terramyacin (31d)- negative: 1/1 |
|  | Enrofloxacin (21d), chloramphenicol (9d), terramyacin (31d)- positive: 1/1 |
|  | Enrofloxacin (21d), chloramphenicol (12d), terramyacin (33d)- positive: 1/1 |
|  | Unknown treatment and duration- negative: 5/5 |
| Outcomes | Chloramphenicol (14d)- released: 1/3; euthanised: 1/3; permanent captive: 1/3 |
|  | Chloramphenicol (21d)- released: 1/1 |
|  | Chloramphenicol (28d)- released: 5/6; unspecified: 1/6 |
|  | Chloramphenicol (unknown)- unspecified: 2/2 |
|  | Doxycycline (14d)- released: 1/1 |
|  | Doxycycline (18d)- released: 1/1 |
|  | Doxycycline (21d)- released: 6/6 |
|  | Doxycycline (28d)- released: 2/5; euthanised: 3/5 |
|  | Doxycycline (35d)- released: 3/3 |
|  | Doxycycline (42d)- released: 1/2; unspecified: 1/2 |
|  | Doxycycline (unknown)- unspecified: 2/2 |
|  | Enrofloxacin (20d), chloramphenicol (22d), chlorsig ointment (17d), terramyacin (31d)- released: 1/1 |
|  | Enrofloxacin (21d), chloramphenicol (9d), terramyacin (31d)- euthanised: 1/1 |
|  | Enrofloxacin (21d), chloramphenicol (12d), terramyacin (33d)- euthanised: 1/1 |
|  | Unknown treatment and duration- unspecified: 5/5 |
| **Treated twice (n=4)** | |
| Adult (2 yrs+) | 4 |
| Juvenile (<2 yrs old) | 0 |
| Unknown (age) | 0 |
| Sex | M: 1; F: 3 |
| Unknown (sex) | 0 |
| Wild/captive | W: 4; C:0 |
| Location | NSW: 2; QLD: 2 |
| Clinical signs present | Y: 4 (UGT: 3; ocular, UGT: 1); N: 0 |
| Treatment and duration | Chloramphenicol (28d): 1 |
|  | Chloramphenicol (28d), chloramphenicol (28d): 1 |
|  | Chloramphenicol (28d), enrofloxacin (7d): 1 |
|  | Doxycycline (21d): 1 |
|  | Doxycycline (28d): 2 |
| PCR results | Chloramphenicol (28d)- negative: 1/1 |
|  | Chloramphenicol (28d), chloramphenicol (28d)- negative: 1/1 |
|  | Chloramphenicol (28d), enrofloxacin (7d)- negative: 1/1 |
|  | Doxycycline (21d)- negative: 1/1 |
|  | Doxycycline (28d)- negative: 2/2 |
| Outcomes | Chloramphenicol (28d)- released: 1/1 |
|  | Chloramphenicol (28d), chloramphenicol (28d)- released: 1/1 |
|  | Chloramphenicol (28d), enrofloxacin (7d)- dead: 1/1 |
|  | Doxycycline (21d)- released: 1/1 |
|  | Doxycycline (28d)- released: 1/2; euthanised: 1/2 |

**Table S3.** Raw data summary of the koalas admitted to wildlife hospitals in 2019.

| **Treated once (n=55)** | |
| --- | --- |
| Adult (2 yrs+) | 50 |
| Juvenile (<2 yrs old) | 4 |
| Unknown (age) | 1 |
| Sex | M: 30; F: 23 |
| Unknown (sex) | 2 |
| Wild/captive | W: 55; C: 0 |
| Location | NSW: 32; QLD: 10; SA: 13 |
| Clinical signs present | Y: 31 (ocular: 18; UGT: 12; both: 1); N: 24; Unknown: 0 |
| Treatment and duration | Chloramphenicol (14d): 1 |
|  | Chloramphenicol (19d): 1 |
|  | Chloramphenicol (28d): 10 |
|  | Chloramphenicol (unknown): 3 |
|  | Doxycycline (4d): 1 |
|  | Doxycycline (21d): 12 |
|  | Doxycycline (28d): 11 |
|  | Doxycycline (35d): 1 |
|  | Doxycycline (unknown): 6 |
|  | Unknown treatment and duration: 9 |
| PCR results | Chloramphenicol (14d)- negative: 1/1 |
|  | Chloramphenicol (19d)- positive: 1/1 |
|  | Chloramphenicol (28d)- negative: 7/10; suspect: 2/10; positive: 1/10 |
|  | Chloramphenicol (unknown)- negative: 2/3; positive: 1/3 |
|  | Doxycycline (4d)- negative: 1/1 |
|  | Doxycycline (21d)- negative: 10/12; positive: 1/12; suspect: 1/12 |
|  | Doxycycline (28d)- negative: 11/11 |
|  | Doxycycline (35d)- negative: 1/1 |
|  | Doxycycline (unknown)- negative: 5/6; suspect: 1/6 |
|  | Unknown treatment and duration- negative: 8/9; suspect: 1/9 |
| Outcomes | Chloramphenicol (14d)- euthanised: 1/1 |
|  | Chloramphenicol (19d)- euthanised: 1/1 |
|  | Chloramphenicol (28d)- released: 9/10; euthanised: 1/10 |
|  | Chloramphenicol (unknown)- released: 2/3; unspecified: 1/3 |
|  | Doxycycline (4d)- released: 1/1 |
|  | Doxycycline (21d)- released: 9/12; euthanised: 2/12; permanent captive: 1/12 |
|  | Doxycycline (28d)- released: 10/11; euthanised: 1/11 |
|  | Doxycycline (35d)- released: 1/1 |
|  | Doxycycline (unknown)- released: 1/6; unspecified: 5/6 |
|  | Unknown treatment and duration- unspecified: 9/9 |
| **Treated twice (n=3)** | |
| Adult (2 yrs+) | 1 |
| Juvenile (<2 yrs old) | 2 |
| Unknown (age) | 0 |
| Sex | M: 1; F: 2 |
| Unknown (sex) | 0 |
| Wild/captive | W: 3; C: 0 |
| Location | NSW: 2, QLD: 1 |
| Clinical signs present | Y: 2 (ocular); N: 2 |
| Treatment and duration | Chloramphenicol (14d): 1 |
|  | Chloramphenicol (28d): 1 |
|  | Doxycycline (21d), doxycycline (21d): 2 |
| PCR results | Chloramphenicol (14d)- negative: 1/1 |
|  | Chloramphenicol (28d)- negative: 1/1 |
|  | Doxycycline (21d), doxycycline (21d)- negative: 2/2 |
| Outcomes | Chloramphenicol (14d)- released: 1/1 |
|  | Chloramphenicol (28d)- released: 1/1 |
|  | Doxycycline (21d), doxycycline (21d)- released: 2/2 |
| **Treated more than twice (n=2)** | |
| Adult (2 yrs+) | 2 |
| Juvenile (<2 yrs old) | 0 |
| Unknown (age) | 0 |
| Sex | M: 1; F: 1 |
| Unknown (sex) | 0 |
| Wild/captive | W: 2; C: 0 |
| Location | NSW: 2 |
| Clinical signs present | Y: 2 (ocular: 1; UGT: 1); N: 0 |
| Treatment and duration | Chloramphenicol (38d), doxycycline (11d), doxycycline (28d): 1 |
|  | Unknown (14d), chloramphenicol (unknown), chloramphenicol (unknown), chloramphenicol (unknown): 1 |
| PCR results | Chloramphenicol (38d), doxycycline (11d), doxycycline (28d)- negative: 1/1 |
|  | Unknown (14d), chloramphenicol (unknown), chloramphenicol (unknown), chloramphenicol (unknown)- negative: 1/1 |
| Outcomes | Chloramphenicol (38d), doxycycline (11d), doxycycline (28d)- released: 1/1 |
|  | Unknown (14d), chloramphenicol (unknown), chloramphenicol (unknown), chloramphenicol (unknown)- unspecified: 1/1 |

**Table S4.** Raw data summary of the koalas admitted to wildlife hospitals in 2020.

| **Treated once (n=26)** | |
| --- | --- |
| Adult (2 yrs+) | 23 |
| Juvenile (<2 yrs old) | 2 |
| Unknown (age) | 1 |
| Sex | M: 12; F: 14 |
| Unknown (sex) | 0 |
| Wild/captive | W: 25; C: 1 |
| Location | NSW: 21; SA: 4; QLD: 1 |
| Clinical signs present | Y: 17 (ocular: 6; UGT: 5; both: 6); N: 8; Unknown: 1 |
| Treatment and duration | Chloramphenicol (25d): 1 |
|  | Chloramphenicol (28d): 3 |
|  | Chloramphenicol (unknown), clavulox (unknown): 1 |
|  | Chloramphenicol (unknown): 1 |
|  | Doxycycline (21d): 1 |
|  | Doxycycline (28d): 5 |
|  | Doxycycline (35d): 1 |
|  | Doxycycline (unknown): 1 |
|  | Enrofloxacin (24d), topical chloramphenicol: 1 |
|  | Tricin (3d): 1 |
|  | Tramadol (unknown): 1 |
|  | Rimadyl injection (1 dose), doxycycline (28d): 1 |
|  | Topical chloramphenicol (4d), enrofloxacin (6d), doxycycline (28d): 1 |
|  | Unknown treatment and duration: 7 |
| PCR results | Chloramphenicol (25d)- suspect: 1/1 |
|  | Chloramphenicol (28d)- negative: 3/3 |
|  | Chloramphenicol (unknown), clavulox (unknown)- negative: 1/1 |
|  | Chloramphenicol (unknown)- negative: 1/1 |
|  | Doxycycline (21d)- negative: 1/1 |
|  | Doxycycline (28d)- negative: 4/5; positive: 1/5 |
|  | Doxycycline (35d)- negative: 1/1 |
|  | Doxycycline (unknown)- negative: 1/1 |
|  | Enrofloxacin (24d), topical chloramphenicol- negative: 1/1 |
|  | Tricin (3d)- negative: 1/1 |
|  | Tramadol (unknown)- negative: 1/1 |
|  | Rimadyl injection (1 dose), doxycycline (28d)- negative: 1/1 |
|  | Topical chloramphenicol (4d), enrofloxacin (6d), doxycycline (28d)- negative: 1/1 |
|  | Unknown treatment and duration- negative: 7/7 |
| Outcomes | Chloramphenicol (25d)- released: 1/1 |
|  | Chloramphenicol (28d)- released: 2/3; euthanised: 1/3 |
|  | Chloramphenicol (unknown), clavulox (unknown)- unspecified: 1/1 |
|  | Chloramphenicol (unknown)- unspecified: 1/1 |
|  | Doxycycline (21d)- released: 1/1 |
|  | Doxycycline (28d)- released: 4/5; escaped: 1/5 |
|  | Doxycycline (35d)- released: 1/1 |
|  | Doxycycline (unknown)- unspecified: 1/1 |
|  | Enrofloxacin (24d), topical chloramphenicol- unspecified: 1/1 |
|  | Tricin (3d)- unspecified: 1/1 |
|  | Tramadol (unknown)- released: 1/1 |
|  | Rimadyl injection (1 dose), doxycycline (28d)- released: 1/1 |
|  | Topical chloramphenicol (4d), enrofloxacin (6d), doxycycline (28d)- released: 1/1 |
|  | Unknown treatment and duration- released: 1/7; unspecified: 6/7 |
| **Treated twice (n=2)** | |
| Adult (2 yrs+) | 1 |
| Juvenile (<2 yrs old) | 1 |
| Unknown (age) | 0 |
| Sex | M: 1; F: 1 |
| Unknown (sex) | 0 |
| Wild/captive | W: 2 |
| Location | NSW: 2 |
| Clinical signs present | Y: 2 (UGT) |
| Treatment and duration | Enrofloxacin (14d), chloramphenicol (14d): 2 |
| PCR results | Enrofloxacin (14d), chloramphenicol (14d)- negative: 2/2 |
| Outcomes | Enrofloxacin (14d), chloramphenicol (14d)- released: 2/2 |
| **Treated more than twice (n=2)** | |
| Adult (2 yrs+) | 1 |
| Juvenile (<2 yrs old) | 0 |
| Unknown (age) | 1 |
| Sex | M: 1; F:1 |
| Unknown (sex) | 0 |
| Wild/captive | W: 2 |
| Location | NSW: 2 |
| Clinical signs present | Y: 2 (ocular: 1; ocular, UGT: 1) |
| Treatment and duration | Topical chloramphenicol (8d), enrofloxacin (14d), chloramphenicol (4d), enrofloxacin (14d): 1 |
|  | Chloramphenicol (30d), neocort and tryolab cream (5d), clavulox and flamazine cream (7d), enrofloxacin (7d), lamsil cream (14d), doxycycline (1 dose): 1 |
| PCR results | Topical chloramphenicol (8d), enrofloxacin (14d), chloramphenicol (4d), enrofloxacin (14d)- negative: 1/1 |
|  | Chloramphenicol (30d), neocort and tryolab cream (5d), clavulox and flamazine cream (7d), enrofloxacin (7d), lamsil cream (14d), doxycycline (1 dose)- negative: 1/1 |
| Outcomes | Topical chloramphenicol (8d), enrofloxacin (14d), chloramphenicol (4d), enrofloxacin (14d)- released: 1/1 |
|  | Chloramphenicol (30d), neocort and tryolab cream (5d), clavulox and flamazine cream (7d), enrofloxacin (7d), lamsil cream (14d), doxycycline (1 dose)- released: 1/1 |

**Table S5.** Raw data of the individual koalas admitted to wildlife hospitals in 2017.

| **Treated once (n=35)** | **Euthanised** | **Ages** | **Sex** | **Wild/captive** | **Location** | **Clinical signs** | **Treatment** | **Length of treatment (days)** | **PCR results (pre-Tx)** | **PCR results (post-Tx)** | **Outcome** | **Length between treatments** |
| --- | --- | --- | --- | --- | --- | --- | --- | --- | --- | --- | --- | --- |
| K1 | No | 6-8 | F | W | SA | Eyes, UGT | Chloramphenicol | 28 | N/A | Negative (eyes, UGT) | Released |  |
| K2 | No | 4 | F | W | QLD | UGT | Chloramphenicol | 28 | N/A | Negative (UGT) | Permanent captive |  |
| K3 | No | 3 | F | W | QLD | UGT | Chloramphenicol | 28 | N/A | Negative (UGT) | Released |  |
| K4 | No | 4 | F | W | QLD | UGT | Chloramphenicol | 28 | Negative (urine) | Negative (UGT) | Released |  |
| K5 | No | 7 | M | W | NSW | No | Chloramphenicol | 45 | N/A | Negative (eyes, UGT) | Released |  |
| K6 | No | 4 | M | W | NSW | Eyes | Chloramphenicol | 28 | N/A | Negative (eyes) | Released |  |
| K7 | No | 3 | F | W | QLD | UGT | Chloramphenicol | 28 | N/A | Negative (UGT) | Pre-released |  |
| K8 | No | 8 | M | W | QLD | Eyes | Chloramphenicol | 28 | N/A | Negative (eyes) | Released |  |
| K9 | No | 2-3 | F | W | NSW | Eyes | Chloramphenicol | 28 | Positive (eyes, UGT) | Negative (eyes, UGT, PHX) | Released |  |
| K10 | No | 3 | M | W | NSW | No | Chloramphenicol | 28 | Positive (eyes, UGT) | Negative (eyes, UGT) | Released |  |
| K11 | No | 3 | F | W | NSW | Eyes | Chloramphenicol | 28 | Positive (eyes, UGT) | Negative (eyes, UGT) | Released |  |
| K12 | No | 5 | M | W | NSW | UGT | Chloramphenicol | 28 | Negative (eyes, PHX); positive (UGT) | Negative (eyes, UGT) | Released |  |
| K13 | No | 6 | M | W | NSW | N/A | Chloramphenicol | N/A | Positive (eyes); negative (UGT) | Negative (eyes, UGT) | Released |  |
| K14 | Yes | 4 | F | W | NSW | UGT | Enrofloxacin, chloramphenicol | Enrofloxacin: 14; chloramphenicol: 21 | N/A | Positive (eyes, UGT) | Euthanised |  |
| K15 | No | 4 | F | W | NSW | Eyes, UGT | Chloramphenicol |  | N/A | Negative (UGT) | Unspecified |  |
| K16 | No | 5 | M | W | NSW | Eyes | Chloramphenicol |  | Positive (eyes) | Negative (eyes) | Unspecified |  |
| K17 | No | 1-2 | F | W | NSW | PHX | N/A | N/A | Positive (PHX) | Negative (PHX) | Permanent captive |  |
| K18 | No | 4 | F | W | NSW | Eyes | Chloramphenicol | 14 | Positive (eyes, UGT) | Negative (eyes, UGT) | Unspecified |  |
| K19 | No | 4 | M | W | QLD | Eyes, UGT | Chloramphenicol | 28 | Negative (eyes); positive (UGT) | Negative (UGT) | Released |  |
| K20 | No | N/A | F | W | NSW | Eyes | Chloramphenicol | 35 | Positive (eyes, UGT) | Suspect (eyes); negative (UGT) | Unspecified |  |
| K21 | No | 2-3 | F | W | NSW | UGT | Chloramphenicol | 28 | Positive (eyes, UGT); negative (PHX) | Positive (eyes, UGT); negative (PHX) | Released |  |
| K22 | No | 4 | F | W | NSW | UGT | Chloramphenicol | 45 | N/A | Negative (eyes, UGT, PHX) | Released |  |
| K23 | No | N/A | F | W | NSW | N/A | Chloramphenicol |  | Positive (eyes) | Negative (eyes, UGT) | Unspecified |  |
| K24 | No | N/A | M | W | NSW | N/A | Chloramphenicol |  | N/A | Negative (eyes, UGT) | Unspecified |  |
| K25 | No | 10-12 | F | W | NSW | Eyes | Chloramphenicol, Chlorsig ointment | 35, 70 | N/A | Negative (eyes, UGT) | Permanent captive |  |
| K26 | No | 4 | F | W | NSW | Eyes | Chloramphenicol | 45 | N/A | Negative (eyes) | Released |  |
| K27 | No | 5-6 | M | W | NSW | Eyes | Chloramphenicol | 28 | N/A | Negative (eyes, UGT, PHX) | Released |  |
| K28 | No | 2-3 | M | W | NSW | Eyes | Chloramphenicol | 45 | N/A | Negative (eyes, UGT, PHX) | Released |  |
| K29 | No | 2-3 | F | W | NSW | UGT | Chloramphenicol |  | Negative (eyes); positive (UGT) | Negative (eyes); suspect (UGT) | Unspecified |  |
| K30 | No | 2-3 | F | W | NSW | Eyes | Chloramphenicol | 28 | N/A | Negative (eyes, UGT, PHX) | Released |  |
| K31 | No | 2-3 | F | W | NSW | Eyes, UGT | Chloramphenicol |  | Positive (eyes, UGT) | Suspect (eyes); negative (UGT) | Unspecified |  |
| K32 | No | 4 | M | W | NSW | Eyes | Chloramphenicol | 28 | Positive (eyes) | Negative (eyes, UGT, PHX) | Released |  |
| K33 | No | 10-12 | M | W | NSW | Eyes | Chloramphenicol | 28 | Suspect (eyes); negative (UGT, PHX) | Negative (eyes, UGT, PHX) | Released |  |
| K34 | No | 4 | M | W | NSW | Eyes, UGT | Chloramphenicol | 28 | Positive (eyes) | Negative (eyes, UGT) | Released |  |
| K35 | Yes | 1-2 | F | W | NSW | Eyes, UGT | Chloramphenicol | 17 | Positive (eyes, UGT) | N/A | Euthanised |  |
| **Treated twice**  **(n=6)** | **Euthanised** | **Ages** | **Sex** | **Wild/captive** | **Location** | **Clinical signs** | **Treatment** | **Length of treatment (days)** | **PCR results (pre-Tx)** | **PCR results (post-Tx)** | **Outcome** | **Length between treatments** |
| K36 | No | 1-2 | F | W | NSW | No | Chloramphenicol | 28 | Positive (eyes) | Positive (PHX); negative (eyes, UGT) | Re-treated | 1 month |
| K36 | Yes | 1-2 | F | W | NSW | Eyes | Chloramphenicol | 21 |  | Suspect (eyes); positive (UGT, PHX) | Euthanised |  |
| K37 | No | 6 | M | W | QLD | Eyes, UGT | Chloramphenicol | 28 | Positive (UGT) | Positive (eyes, UGT) | Re-treated | 1 month |
| K37 | No | 6 | M | W | QLD | No | Chloramphenicol | 14 |  | Negative (eyes, UGT) | Unspecified |  |
| K38 | No | 4 | M | W | NSW | UGT | Chloramphenicol |  | N/A | Positive (eyes, UGT) | Re-treated | >1 month |
| K38 | No | 4 | M | W | NSW | UGT | Chloramphenicol |  |  | Negative (eyes, UGT) | Unspecified |  |
| K39 | No | 9 | F | W | NSW | UGT | Chloramphenicol | 28 | N/A | Negative (eyes, UGT, PHX) | Re-treated | 1 month |
| K39 | Yes | 9 | F | W | NSW | UGT | N/A | N/A |  | Negative (UGT) | Euthanised |  |
| K40 | No | 5 | M | W | NSW | No | Chloramphenicol | 42 | N/A | Suspect (eyes); Negative (UGT, PHX) | Released | 3 months |
| K40 | No | 5 | M | W | NSW | No | N/A | N/A |  | Negative (eyes, UGT, PHX) | Released |  |
| K41 | No | 4 | M | W | NSW | UGT | Chloramphenicol | 28 | Positive (eyes, UGT) | Negative (eyes), positive (UGT) | Re-treated | Continuous |
| K41 | No | 4 | M | W | NSW | UGT | Chloramphenicol | 7 |  | Negative (eyes, UGT) | Released |  |

**Table S6.** Raw data of the individual koalas admitted to wildlife hospitals in 2018.

| **Treated once (n=40)** | **Euthanised** | **Ages** | **Sex** | **Wild/captive** | **Location** | **Clinical signs** | **Treatment** | **Length of treatment (days)** | **PCR results (pre-Tx)** | **PCR results (post-Tx)** | **Outcome** | **Length between treatments** |
| --- | --- | --- | --- | --- | --- | --- | --- | --- | --- | --- | --- | --- |
| K42 | No | N/A | F | W | NSW | Eyes |  |  | N/A | Negative (eyes, UGT) | Unspecified |  |
| K43 | No | N/A | M | W | NSW | Eyes | Chloramphenicol | 28 | Positive (eyes, UGT) | Negative (eyes, UGT) | Unspecified |  |
| K44 | No | 4 | F | W | SA | Eyes | Doxycycline | 21 | Negative (eyes); positive (UGT) | Negative (eyes, UGT) | Released |  |
| K45 | No | 2-3 | F | W | SA | UGT |  |  | Negative (eyes); suspect (UGT) | Negative (eyes, UGT) | Unspecified |  |
| K46 | No | 2-3 | M | W | NSW | No | Chloramphenicol | 28 | Positive (eyes) | Negative (eyes, UGT, PHX) | Released |  |
| K47 | No | 5-6 | M | W | NSW | No | Doxycycline | 42 | Positive (UGT) | Negative (eyes, UGT, PHX) | Unspecified |  |
| K48 | Yes | 5-6 | M | W | NSW | UGT | Chloramphenicol | 14 | Positive (eyes, UGT) | Negative (eyes, UGT, PHX) | Euthanised |  |
| K49 | No | 5-6 | M | W | NSW | UGT | Doxycycline, QUT chlamydial vaccine | 28 | N/A | Negative (eyes, UGT, PHX) | Released |  |
| K50 | No | 2-3 | F | W | NSW | Eyes, UGT | Chloramphenicol | 21 | Positive (eyes) | Negative (eyes, UGT, PHX) | Released |  |
| K51 | No | 5-6 | F | W | NSW | UGT | Doxycycline | 35 | Positive (eyes, UGT) | Negative (eyes, UGT, PHX) | Released |  |
| K52 | No | 5-6 | M | W | QLD | Eyes | Chloramphenicol | 28 | Positive (eyes) | Negative (eyes) | Released |  |
| K53 | No | 8 | F | W | SA | Eyes, UGT | Doxycycline | 21 | Negative (eyes); positive (UGT) | Negative (eyes, UGT) | Released |  |
| K54 | No | N/A | F | W | SA | No | Doxycycline |  | N/A | Negative (eyes, UGT) | Unspecified |  |
| K55 | No | 1 | M | W | SA | No | Doxycycline | 21 | Negative (eyes); positive (UGT) | Negative (eyes, UGT) | Released |  |
| K56 | Yes | 4 | F | W | SA | Eyes | Doxycycline | 28 | Negative (eyes); suspect (UGT) | Negative (eyes, UGT) | Euthanised (renal failure) |  |
| K57 | No | 1-2 | M | W | NSW | No | Doxycycline | 21 | Negative (eyes, UGT, PHX) | Negative (eyes, UGT, PHX) | Released |  |
| K58 | No | 1-2 | M | W | NSW | Eyes | Doxycycline | 21 | Positive (eyes); negative (UGT, PHX) | Negative (eyes, UGT, PHX) | Released |  |
| K59 | No | 12+ | M | W | NSW | Eyes | Chloramphenicol |  | Positive (eyes, UGT) | Suspect (eyes); Negative (UGT) | Unspecified |  |
| K60 | No | 4 | M | W | QLD | Eyes | Chloramphenicol | 28 | N/A | Negative (eyes) | Released |  |
| K61 | No | 5-6 | M | W | NSW | No | Doxycycline | 42 | Positive (eyes) | Negative (eyes, UGT, PHX) | Released |  |
| K62 | No | 4 | F | W | QLD | Eyes | Chloramphenicol | 28 | Positive (eyes); negative (UGT) | Negative (eyes, UGT) | Released |  |
| K63 | No | 5-6 | M | W | QLD | UGT | Chloramphenicol | 14 | Positive (UGT) | Negative (UGT) | Pre-released |  |
| K64 | No | 2-3 | M | W | NSW | No |  |  | Negative (eyes); positive (UGT) | Negative (eyes, UGT) | Unspecified |  |
| K65 | No | 2-3 | M | W | NSW | No |  |  | N/A | Negative (eyes, UGT) | Unspecified |  |
| K66 | No | 4 | F | W | QLD | No | Chloramphenicol | 14 | Positive (UGT) | Negative (UGT) | Permanent captive |  |
| K67 | No | 2-3 | M | W | NSW | No | Doxycycline | 28 | Positive (eyes, UGT) | Negative (eyes, UGT, PHX) | Released |  |
| K68 | No | 10-12 | M | W | NSW | Eyes |  |  | Positive (eyes, UGT) | Negative (eyes, UGT) | Unspecified |  |
| K69 | Yes | 5-6 | M | W | NSW | Eyes | Doxycycline | 28 | Positive (eyes) | Negative (eyes, UGT) | Euthanised |  |
| K70 | No | 5-6 | M | W | QLD | Eyes | Chloramphenicol | 28 | Positive (eyes) | Negative (eyes, UGT) | Released |  |
| K71 | No | 1-2 | M | W | NSW | No | Doxycycline | 21 | Positive (eyes, UGT) | Negative (eyes, UGT) | Released |  |
| K72 | No | 10-12 | M | C | WA | UGT | Doxycycline |  | N/A | Negative (eyes, UGT) | Unspecified |  |
| K73 | No | 4 | F | W | NSW | Eyes | Enrofloxacin, chloramphenicol, chlorsig ointment, terramyacin ointment | 20, 22, 17, 32 | N/A | Negative (UGT) | Released |  |
| K74 | Yes | 10-12 | M | W | NSW | Eyes | Enrofloxacin, chloramphenicol, terramyacin ointment | 21, 9, 31 | N/A | Positive (eyes, UGT) | Euthanised |  |
| K75 | Yes | 5-6 | F | W | NSW | Eyes | Enrofloxacin, chloramphenicol, terramyacin ointment | 21, 12, 33 | N/A | Positive (eyes, UGT) | Euthanised |  |
| K76 | No | 5-6 | F | W | NSW | Eyes | Doxycycline | 14 | Positive (eyes); negative (UGT) | Negative (eyes, UGT) | Released |  |
| K77 | No | 10-12 | F | C | NSW | UGT | Chloramphenicol |  | Positive (UGT) | Negative (eyes); Positive (UGT) | Unspecified |  |
| K78 | Yes | 4 | F | W | NSW | UGT | Doxycycline | 28 | Positive (eyes, UGT) | Positive (UGT) | Euthanised |  |
| K79 | No | 5-6 | M | W | NSW | No | Doxycycline | 35 | Negative (eyes); positive (UGT) | Negative (eyes, UGT) | Released |  |
| K80 | No | 2-3 | M | W | NSW | UGT | Doxycycline | 35 | Suspect (eyes); positive (UGT) | Negative (eyes, UGT) | Released |  |
| K81 | No | 2-3 | F | W | SA | UGT | Doxycycline | 18 | Negative (eyes); suspect (UGT) | Negative (eyes, UGT) | Released |  |
| **Treated twice**  **(n=4)** | **Euthanised** | **Ages** | **Sex** | **Wild/captive** | **Location** | **Clinical signs** | **Treatment** | **Length of treatment (days)** | **PCR results (pre-Tx)** | **PCR results (post-Tx)** | **Outcome** | **Length between treatments** |
| K82 | No | 5-6 | F | W | NSW | UGT | Doxycycline | 21 | N/A | Negative (eyes, UGT, PHX) | Released | 3 months |
| K82 | Yes | 5-6 | F | W | NSW | UGT | Doxycycline | 28 | Positive (UGT) | Negative (UGT) | Euthanised |  |
| K83 | No | 10-12 | F | W | QLD | N/A | Chloramphenicol | 28 | N/A | Positive (UGT) | Re-treated | Continuous |
| K83 | No | 10-12 | F | W | QLD | UGT | Chloramphenicol | 28 |  | Negative (UGT) | Released |  |
| K84 | No | 10-12 | F | W | QLD | UGT | Doxycycline | 28 | Positive (UGT) | Negative (eyes, UGT) | Released | 8 months |
| K84 | No | 10-12 | F | W | QLD | UGT | Chloramphenicol | 28 | N/A | Negative (eyes, UGT) | Released |  |
| K85 | Yes | 4 | M | W | NSW | No | Chloramphenicol | 28 | Positive (eyes, UGT) | Positive (eyes, UGT) | Re-treated | Continuous |
| K85 | Yes | 4 | M | W | NSW | Eyes, UGT | Enrofloxacin | 7 |  | Negative (eyes, UGT) | Dead |  |

**Table S7.** Raw data of the individual koalas admitted to wildlife hospitals in 2019.

| **Treated once (n=57)** | **Euthanised** | **Ages** | **Sex** | **Wild/captive** | **Location** | **Clinical signs** | **Treatment** | **Length of treatment (days)** | **PCR result (pre-Tx)** | **PCR results (post-Tx)** | **Outcome** | **Length between treatments** |
| --- | --- | --- | --- | --- | --- | --- | --- | --- | --- | --- | --- | --- |
| K86 | No | 5-6 | F | W | QLD | UGT | Chloramphenicol | 28 | Negative (eyes); positive (UGT) | Negative (eyes, UGT) | Released |  |
| K87 | Yes | 4 | M | W | QLD | UGT | Chloramphenicol | 19 | N/A | Suspect (eyes); positive (UGT) | Euthanised |  |
| K88 | No | 5-6 | M | W | NSW | No | Chloramphenicol | 28 | N/A | Negative (eyes, UGT) | Released |  |
| K89 | No | 1-2 | F | W | NSW | No | Doxycycline | 28 | Positive (eyes) | Negative (eyes, UGT, PHX) | Released |  |
| K90 | No | 1-2 | M | W | QLD | Eyes | Chloramphenicol | 28 | N/A | Negative (eyes) | Released |  |
| K91 | No | 10-12 | M | W | NSW | No |  |  | N/A | Negative (eyes, UGT) | Unspecified |  |
| K92 | No | N/A | F | W | NSW | UGT | Doxycycline |  | N/A | Suspect (eyes); negative (UGT) | Unspecified |  |
| K93 | Yes | 5-6 | F | W | QLD | Eyes | Chloramphenicol | 28 | Positive (eyes); negative (UGT) | Positive (eyes) | Euthanised |  |
| K94 | No | 1-2 | F | W | SA | No |  |  | Suspect (eyes); positive (UGT) | Negative (eyes, UGT) | Unspecified |  |
| K95 | No | 4 | F | W | SA | UGT | Doxycycline | 4 | Suspect (eyes); positive (UGT) | Negative (eyes, UGT) | Released |  |
| K96 | No | 4 | F | W | QLD | UGT | Chloramphenicol |  | Positive (UGT) | Negative (UGT) | Pre-released |  |
| K97 | No | 2-3 | M | W | QLD | Eyes | Chloramphenicol | 28 | Positive (eyes); suspect (UGT) | Negative (eyes, UGT) | Released |  |
| K98 | No | 5-6 | M | W | NSW | No | Doxycycline | 21 | Positive (UGT) | Negative (eyes, UGT) | Released |  |
| K99 | No | 4 | F | W | NSW | Eyes | Chloramphenicol |  | Positive (eyes) | Positive (eyes) | Unspecified |  |
| K100 | No | 2-3 | M | W | QLD | No | Chloramphenicol | 28 | Positive (eyes) | Negative (eyes); suspect (UGT) | Released |  |
| K101 | No | 10-12 | F | W | QLD | UGT | Chloramphenicol | 28 | Positive (UGT) | Negative (UGT) | Released |  |
| K102 | No | 1-2 | M | W | QLD | Eyes | Chloramphenicol | 28 | Positive (eyes) | Suspect (eyes) | Released |  |
| K103 | No | 5-6 | M | W | QLD | UGT | Chloramphenicol | 28 | Positive (eyes); negative (UGT) | Negative (UGT) | Released |  |
| K104 | No | 10-12 | F | W | SA | No |  |  | N/A | Negative (UGT) | Unspecified |  |
| K105 | No | 4 | F | W | SA | No | Doxycycline |  | Negative (eyes); positive (UGT) | Negative (eyes, UGT) | Unspecified |  |
| K106 | No | 5-6 | F | W | SA | Eyes | Doxycycline |  | N/A | Negative (eyes, UGT) | Unspecified |  |
| K107 | No | 2-3 | M | W | SA | No |  |  | N/A | Negative (eyes, UGT) | Unspecified |  |
| K108 | No | 4 | M | W | NSW | No | Doxycycline | 21 | Suspect (eyes, UGT) | Negative (eyes, UGT) | Released |  |
| K109 | No | 5-6 | M | W | NSW | No | Chloramphenicol | 28 | N/A | Negative (eyes, UGT) | Released |  |
| K110 | No | 5-6 | M | W | SA | No |  |  | N/A | Negative (eyes, UGT) | Unspecified |  |
| K111 | Yes | 12+ | F | W | NSW | UGT | Doxycycline | 21 | Suspect (UGT) | Negative (eyes, UGT, PHX) | Euthanised |  |
| K112 | No | 10-12 | M | W | NSW | Eyes | Doxycycline | 21 | N/A | Suspect (eyes); negative (UGT) | Released |  |
| K113 | No | 5-6 | N/A | W | NSW | Eyes | Chloramphenicol |  | Positive (eyes) | Negative (eyes, UGT) | Pre-released |  |
| K114 | No | 10-12 | M | W | NSW | UGT |  |  | Positive (eyes) | Negative (eyes, UGT) | Unspecified |  |
| K115 | No | 5-6 | F | W | SA | No |  |  | Suspect (eyes); positive (UGT) | Negative (eyes, UGT) | Unspecified |  |
| K116 | No | 5-6 | M | W | NSW | Eyes | Doxycycline | 28 | Positive (eyes); suspect (UGT) | Negative (eyes, UGT) | Released |  |
| K117 | Yes | 4 | F | W | NSW | Eyes | Chloramphenicol | 14 | N/A | Negative (eyes, UGT, PHX) | Euthanised |  |
| K118 | No | 10-12 | M | W | NSW | Eyes | Doxycycline | 28 | N/A | Negative (eyes, UGT) | Released |  |
| K119 | No | 4 | M | W | NSW | No | Doxycycline | 28 | N/A | Negative (eyes, UGT) | Released |  |
| K120 | No | 10-12 | F | W | NSW | Eyes | Doxycycline | 21 | Positive (eyes); negative (UGT) | Negative (eyes, UGT) | Released |  |
| K121 | Yes | 4 | F | W | NSW | No | Doxycycline | 28 | N/A | Negative (eyes, UGT) | Euthanised |  |
| K122 | No | 5-6 | F | W | NSW | Eyes, UGT | Doxycycline | 21 | N/A | Positive (eyes, UGT) | Permanent captive |  |
| K123 | Yes | 4 | M | W | NSW | No | Doxycycline | 21 | Negative (eyes); positive (UGT) | Negative (UGT) | Euthanised |  |
| K124 | No | 5-6 | M | W | NSW | No | Doxycycline | 21 | N/A | Negative (UGT) | Released |  |
| K125 | No | 5-6 | M | W | SA | No |  |  | Negative (eyes); positive (UGT) | Negative (eyes, UGT) | Unspecified |  |
| K126 | No | 10-12 | M | W | NSW | Eyes | Doxycycline | 35 | N/A | Negative (eyes, UGT) | Released |  |
| K127 | No | 4 | M | W | NSW | Eyes | Doxycycline | 28 | N/A | Negative (eyes, UGT) | Released |  |
| K128 | No | 10-12 | M | W | NSW | No | Doxycycline | 21 | N/A | Negative (eyes, UGT) | Released |  |
| K129 | No | 10-12 | M | W | NSW | Eyes | Doxycycline | 28 | Positive (UGT) | Negative (eyes, UGT) | Released |  |
| K130 | No | 5-6 | M | W | NSW | No | Doxycycline | 28 | N/A | Negative (eyes, UGT) | Released |  |
| K131 | No | 5-6 | N/A | W | SA | Eyes |  |  | Negative (eyes); positive (UGT) | Suspect (eyes); negative (UGT) | Unspecified |  |
| K132 | No | 10-12 | F | W | NSW | No | Doxycycline | 28 | Positive (eyes) | Negative (eyes, UGT) | Released |  |
| K133 | No | 2-3 | F | W | NSW | No | Doxycycline | 21 | Positive (UGT) | Negative (eyes, UGT) | Released |  |
| K134 | No | 5-6 | F | W | SA | UGT | Doxycycline |  | Positive (UGT) | Negative (eyes, UGT) | Unspecified |  |
| K135 | No | 4 | F | W | SA | UGT | Doxycycline |  | Suspect (UGT) | Negative (eyes, UGT) | Unspecified |  |
| K136 | No | 5-6 | F | W | SA | UGT | Doxycycline |  | Positive (UGT) | Negative (eyes, UGT) | Unspecified |  |
| K137 | No | 5-6 | M | W | NSW | Eyes | Doxycycline | 28 | Positive (eyes, UGT) | Negative (eyes, UGT) | Released |  |
| K138 | No | 5-6 | M | W | NSW | No | Doxycycline | 21 | N/A | Negative (eyes, UGT) | Released |  |
| K139 | No | 4 | M | W | NSW | No | Doxycycline | 21 | Positive (UGT) | Negative (eyes, UGT) | Released |  |
| K140 | No | 5-6 | M | W | NSW | Eyes | Doxycycline | 28 | N/A | Negative (eyes, UGT) | Released |  |
| **Treated twice (n=3)** | **Euthanised** | **Ages** | **Sex** | **Wild/captive** | **Location** | **Clinical signs** | **Treatment** | **Length of treatment (days)** | **PCR results (pre-Tx)** | **PCR results (post-Tx)** | **Outcome** | **Length between treatments** |
| K141 | No | 1-2 | M | W | NSW | Eyes | Doxycycline | 21 | Positive (eyes, UGT) | Suspect (eyes, UGT); negative (PHX) | Re-treated | Continuous |
| K141 | No | 1-2 | M | W | NSW | Eyes | Doxycycline | 21 |  | Negative (eyes, UGT) | Released |  |
| K142 | No | 15+ | F | W | NSW | No | Doxycycline | 21 | Positive (eyes) | Positive (eyes); negative (UGT, PHX) | Re-treated | Continuous |
| K142 | No | 15+ | F | W | NSW | No | Doxycycline | 21 |  | Negative (eyes, UGT) | Released |  |
| K143 | No | 1-2 | F | W | QLD | Eyes | Chloramphenicol | 28 | Positive (eyes, UGT) | Negative (eyes, UGT) | Pre-released | 2 months |
| K143 | No | 1-2 | F | W | QLD | No | Chloramphenicol | 14 | Positive (eyes) | Negative (eyes) | Pre-released |  |
| **Treated more than twice**  **(n=2)** | **Euthanised** | **Ages** | **Sex** | **Wild/captive** | **Location** | **Clinical signs** | **Treatment** | **Length of treatment (days)** | **PCR results (pre-Tx)** | **PCR results (post-Tx)** | **Outcome** | **Length between treatments** |
| K144 | No | 5-6 | F | W | NSW | Eyes, UGT | Chloramphenicol | 38 | N/A | Positive (UGT) | Re-treated | Continuous |
| K144 | No | 5-6 | F | W | NSW | UGT | Doxycycline | 11 |  | Negative (UGT) | Re-treated | Continuous |
| K144 | No | 5-6 | F | W | NSW | UGT | Doxycycline | 28 |  | Negative (UGT) | Released |  |
| K145 | No | 2-3 | M | W | NSW | Eyes | Chloramphenicol | 14 | N/A | Positive (eyes, UGT) | Re-treated | Continuous |
| K145 | No | 2-3 | M | W | NSW | Eyes | Chloramphenicol |  |  | Negative (eyes, UGT) | Re-treated | Continuous |
| K145 | No | 2-3 | M | W | NSW | Eyes | Chloramphenicol |  |  | Negative (eyes); Suspect (UGT) | Re-treated (still has mild keratoconjunctivitis) | Continuous |
| K145 | No | 2-3 | M | W | NSW | Eyes | Chloramphenicol |  |  | Negative (eyes, UGT) | Unspecified |  |

**Table S8.** Raw data of the individual koalas admitted to wildlife hospitals in 2020.

| **Treated once**  **(n=27)** | **Euthanised** | **Ages** | **Sex** | **Wild/captive** | **Location** | **Clinical signs** | **Treatment** | **Length of treatment (days)** | **PCR results (pre-Tx)** | **PCR results (post-Tx)** | **Outcome** | **Length between treatments** |
| --- | --- | --- | --- | --- | --- | --- | --- | --- | --- | --- | --- | --- |
| K146 | No | 4 | F | W | NSW | Eyes, UGT |  |  | Positive (eyes, UGT) | Negative (eyes, UGT) | Unspecified |  |
| K147 | No | 4 | F | W | NSW | No | Doxycycline | 28 | N/A | Negative (eyes, UGT) | Released |  |
| K148 | No | 10-12 | M | W | NSW | No | Doxycycline | 28 | Positive (eyes, UGT) | Negative (eyes) | Released |  |
| K149 | No | 4 | F | W | SA | Eyes | Tricin | 3 | Positive (eyes) | Negative (eyes) | Unspecified |  |
| K150 | No | 10-12 | F | W | NSW | Eyes | Chloramphenicol ointment, enrofloxacin, doxycycline | 4, 6, 28 | Positive (eyes, UGT) | Negative (eyes, UGT) | Released |  |
| K151 | No | 5-6 | M | W | NSW | N/A |  |  | Positive (eyes, UGT) | Negative (eyes, UGT) | Unspecified |  |
| K152 | No | 1-2 | F | W | NSW | Eyes, UGT | Chloramphenicol | 28 | Positive (eyes, UGT) | Negative (eyes, UGT) | Pre-released |  |
| K153 | No | 1-2 | M | W | NSW | No | Doxycycline | 35 | Negative (eyes, UGT); positive (PHX) | Negative (eyes, UGT, PHX) | Released |  |
| K154 | Yes | 10-12 | F | W | NSW | Eyes, UGT | Chloramphenicol | 28 | N/A | Negative (eyes, UGT) | Euthanised |  |
| K155 | No | 2-3 | M | W | NSW | No |  |  | N/A | Negative (eyes, UGT) | Unspecified |  |
| K156 | No | 10-12 | F | C | QLD | Eyes, UGT | Enrofloxacin, chloramphenicol (topical) | 24 | Positive (eyes) | Negative (eyes, UGT) | Unspecified |  |
| K157 | No | 5-6 | F | W | SA | UGT | Doxycycline | 28 | Negative (eyes); positive (UGT) | Negative (eyes, UGT) | Released |  |
| K158 | No | 2-3 | M | W | NSW | Eyes, UGT |  |  | N/A | Negative (eyes, UGT) | Unspecified |  |
| K159 | No | 5-6 | F | W | SA | UGT | Doxycycline | 28 | Negative (eyes); positive (UGT) | Negative (eyes, UGT) | Released |  |
| K160 | No | 10-12 | M | W | NSW | Eyes |  |  | Positive (eyes) | Negative (eyes, UGT) | Unspecified |  |
| K161 | No | 10-12 | M | W | NSW | No |  |  | N/A | Negative (eyes, UGT) | Unspecified |  |
| K162 | No | 4 | M | W | NSW | Eyes | Chloramphenicol, clavulox |  | Positive (eyes, UGT) | Negative (eyes, UGT) | Unspecified |  |
| K163 | No | 5-6 | M | W | NSW | UGT | Chloramphenicol | 25 | Negative (eyes); positive (UGT) | Suspect (eyes); negative (UGT) | Pre-released |  |
| K164 | No | 5-6 | M | W | NSW | Eyes | Doxycycline | 21 | Positive (eyes) | Negative (eyes, UGT) | Released |  |
| K165 | No | 5-6 | M | W | SA | UGT | Doxycycline |  | N/A | Negative (UGT) | Unspecified |  |
| K166 | No | 2-3 | F | W | NSW | No | Rimadyl injection, doxycycline | 1 dose, 28 | Negative (eyes); positive (UGT) | Negative (eyes, UGT) | Released |  |
| K167 | No | 2-3 | M | W | NSW | Eyes | Tramadol |  | Positive (eyes, UGT) | Negative (eyes, UGT) | Pre-released |  |
| K168 | No | N/A | F | W | NSW | No |  |  | N/A | Negative (eyes, UGT) | Pre-released |  |
| K169 | No | 5-6 | F | W | NSW | Eyes, UGT | Chloramphenicol |  | Positive (eyes, UGT) | Negative (eyes, UGT) | Unspecified |  |
| K170 | No | 5-6 | F | W | NSW | No | Chloramphenicol | 28 | Positive (eyes, UGT) | Negative (eyes, UGT) | Released |  |
| K171 | No | 2-3 | F | W | NSW | UGT | Doxycycline | 28 | N/A | Suspect (eyes); positive (UGT) | Escaped |  |
| K172 | No | 1-2 | F | W | QLD | Eyes | Doxycycline | 14 | Positive (eyes) | Negative (eyes, UGT) | Permanent captive |  |
| K173 | No | 5-6 | M | W | QLD | No | Doxycycline | 28 | Positive (eyes, UGT) | Negative (eyes, UGT) | Released |  |
| **Treated twice (n=2)** | **Euthanised** | **Ages** | **Sex** | **Wild/captive** | **Location** | **Clinical signs** | **Treatment** | **Length of treatment (days)** | **PCR results (pre-Tx)** | **PCR results (post-Tx)** | **Outcome** | **Length between treatment** |
| K174 | No | 2-3 | M | W | NSW | UGT | Enrofloxacin | 14 | Negative (eyes); positive (UGT) | Positive (UGT) | Re-treated | Continuous |
| K174 | No | 2-3 | M | W | NSW | UGT | Chloramphenicol | 14 |  | Negative (eyes, UGT) | Released |  |
| K175 | No | 1-2 | F | W | NSW | UGT | Enrofloxacin | 14 | Suspect (eyes); positive (UGT) | Positive (UGT) | Re-treated | Continuous |
| K175 | No | 1-2 | F | W | NSW | UGT | Chloramphenicol | 14 |  | Negative (eyes, UGT) | Pre-released |  |
| **Treated more than twice (n=2)** | **Euthanised** | **Ages** | **Sex** | **Wild/captive** | **Location** | **Clinical signs** | **Treatment** | **Length of treatment (days)** | **PCR results (pre-Tx)** | **PCR results (post-Tx)** | **Outcome** | **Length between treatment** |
| K176 | No | 5-6 | M | W | NSW | Eyes, UGT | Chloramphenicol ointment, enrofloxacin | 8, 14 | Negative (eyes); positive (UGT) | Positive (eyes, UGT) | Re-treated | Continuous |
| K176 | No | 5-6 | M | W | NSW | Eyes, UGT | Chloramphenicol | 4 |  | N/A | Re-treated (adverse reaction) | Continuous |
| K176 | No | 5-6 | M | W | NSW | Eyes, UGT | Enrofloxacin | 14 |  | Negative (eyes, UGT) | Released |  |
| K177 | No | N/A | F | W | NSW | UGT | Chloramphenicol | 30 | N/A | N/A | Re-treated (nasal crusting) | Continuous |
| K177 | No | N/A | F | W | NSW | PHX | Neocort, tryolab cream | 5 |  | N/A | Re-treated (eyes discharge) | Continuous |
| K177 | No | N/A | F | W | NSW | Eyes | Clavulox, flamazine cream | 7 |  | Negative (eyes) | Re-treated | Continuous |
| K177 | No | N/A | F | W | NSW | Eyes | Enrofloxacin | 7 |  | N/A | Re-treated | Continuous |
| K177 | No | N/A | F | W | NSW | Eyes | Lamsil cream | 14 |  |  | Re-treated | Continuous |
| K177 | No | N/A | F | W | NSW | Eyes | Doxycycline | 1 dose |  | Negative (eyes, UGT) | Released |  |
